# An iterative process and mixture design approach for dry granulated ternary blends of filler-binders

**DOI:** 10.1016/j.ijpx.2025.100331

**Published:** 2025-04-01

**Authors:** Niclas Märkle, Gernot Warnke, Miriam Pein-Hackelbusch

**Affiliations:** a Ostwestfalen-Lippe University of Applied Sciences and Arts, Institute for Life Science Technologies (ILT.NRW), Campusallee 12, 32657 Lemgo, Germany; b JRS Pharma, Holzmuehle 1, 73494 Rosenberg, Germany

**Keywords:** Design of experiments, Roller compaction/dry granulation, RC/DG, Dicalcium phosphate, Microcrystalline cellulose, Silicified microcrystalline cellulose

## Abstract

Roller compaction/dry granulation (RC/DG) is a key process in pharmaceutical manufacturing for improving powder flowability, density, and segregation resistance. Advanced statistical modeling was used to optimize RC/DG process parameters and subsequently binder compositions by employing process and mixture design experiments. The authors used microcrystalline cellulose (MCC), silicified MCC (SMCC), and dicalcium phosphate (DCP) as filler-binder examples in RC/DG experiments. Granule and tablet properties, including flowability, bulk and tapped densities, as well as resistance to crushing, were analyzed using compendial methods. The process design experiments confirmed that RC/DG reduces manufacturability compared to direct compression. Optimal processing conditions, balancing sufficient tablet strengths and granule formation, were identified to be between 20 (SCF * ϑ) [kN/cm] and ∼ 60 (SCF * ϑ) [kN/cm]. Thereby (ϑ) is defined as the screw-to-roll speed ratio and (SFC) as the specific compaction force. Mixture design experiments revealed optimal mixtures balancing SMCC, MCC, and DCP to achieve desired properties like low angle of repose, high bulk density, and strong tablets. These findings provide guidance for selecting formulations and process parameters in RC/DG applications. The derived ‘SCF * ϑ’- factor was found to effectively describe the granulation intensity. A superimposed mixture design model based on precise target values of the parameters bulk density, flow properties, and breaking force allowed identification of the best formulation.

## Highlights

• An iterative design for process- mixture-design optimization is presented.
• Optimal process settings for RC/DG are identified based on the ‘SCF * ϑ’ factor.
• Following ICH Q8, a model is built based on combined target parameters.

## Graphical abstract

**Figure:**
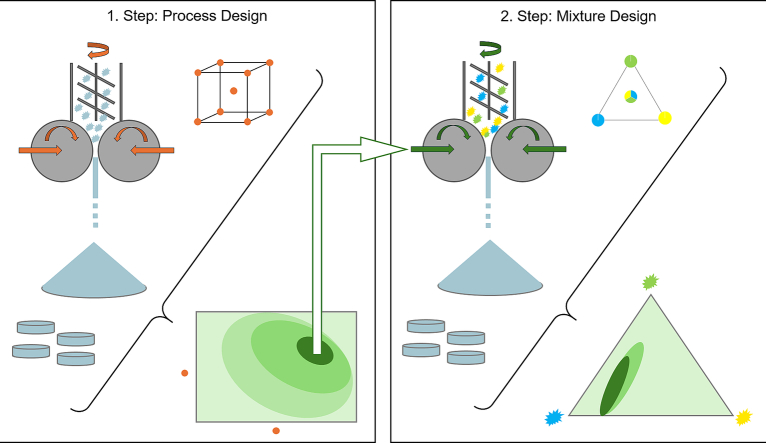

## 1 Introduction

In many processes in the field of pharmaceutical technology, powdered raw and intermediate products are used. In the interest of uncomplicated processing, the primary particles are often initially agglomerated into granules. Thereby, material is produced, which has a reduced tendency to segregate (Keitzer, 2021), and which exhibits improved flow characteristics. The latter is achieved via a narrow -ideally monomodal- particle size distribution and a reduced bulk volume, thus decreasing the specific surface area and the particle-particle interaction (Schiano et al., 2016).

To achieve mentioned benefits, various processes, relating to wet, melt and dry granulation, can be applied. Thereof, wet granulation is mainly used in pharmaceutical industry (Thapa et al., 2019). However, the required drying step can initiate degradation processes of heat sensitive substances, and is time, and thus, cost intensive. Now, compared to three types of wet granulators (a fluidized bed granulator, a high shear granulator and a twin screw granulator) – it was recently shown that a roller compactor used for dry granulation was the most efficient with regard to energy and time (Karunanayake et al., 2024). The underlying roller compaction/dry granulation (RC/DG) process is furthermore priorised over wet granulation by the manufacturing classification system for oral solid dosage forms, when direct compression is not feasible (Leane et al., 2015). Consequently, RC/DG has become a standard technique in pharmaceutical manufacturing (Kleinebudde, 2022). Gaining knowledge about process characteristics and material behavior is, therefore, of growing interest. In line with this, many studies have evaluated the impact of the critical process parameters, such as the specific compaction force SCF [kN/cm], the roll gap width [cm], the roll speed N_R_ [min^−1^], and the feeding screw speed N_S_ [min^−1^], alone or in combination, on the quality of the ribbons, granules and relating tablets.

Since ribbons with a high solid fraction lead to coarser granules (Jaminet and Hess, 1966) and improved flowability (Wagner et al., 2013), processes are designed to produce according ribbons. Researchers found that SCF has the highest impact on the ribbon's solid fraction with an increased SCF leading to an increased solid fraction of the produced ribbons (Csordas et al., 2018), which also increased ribbon tensile strength (Reimer and Kleinebudde, 2019). It has also been shown that SCF is directly linked to the granule size distribution as an increase in SCF leads to a decreased fraction of fines (Mangal and Kleinebudde, 2018). By increasing the roll gap width at constant SCF, ribbons with increased thickness and decreased relative densities were produced (Peter et al., 2010). Also N_R_ impacts the density and tensile strength of the ribbons (Atanaskova et al., 2020; Kleinebudde, 2022; Li et al., 2024; Rowe et al., 2017; Souihi et al., 2015). However, the impact of N_R_ on ribbon solid fraction is controversally discussed in the literature. This is why (Lück et al., 2022) systematically investigated the influence of N_R_ at different SCF for different materials on ribbon and granule properties. Their results indicated that the solid fraction of ribbons made from plastic materials is, compared to the solid fraction of ribbons made from brittle materials, more affected by N_R_. Recently also different working groups (Li et al., 2024; Muthancheri et al., 2024) developed roller compaction models accounting for the importance of N_R_ on the product quality. (Li et al., 2024) thereby improved the model of (Johanson, 1965) and found that the ribbon solid fraction depends on both, N_R_ and the composition of the formulation. (Muthancheri et al., 2024) introduced a modification of the model of (Sousa et al., 2020) which particularly improved the prediction accuracy, particularly at higher N_R_.

If tablets are to be produced based on RC/DG material, their tensile strengths are strongly influenced by the properties of the ribbons and related granules (Boersen et al., 2015). Particularly, the work hardening phenomenon (Malkowska and Khan, 1983), related to a loss in tabletability, has to be taken into consideration (Sun and Kleinebudde, 2016).

It becomes obvious that optimizing the RC/DG process is complex. Since many factors interact, their impact on the quality of the final product is often difficult to predict. Design of Experiments (DoE) is a fundamental tool (Politis et al., 2017) for systematically investigating and optimizing a roller compaction process (Atanaskova et al., 2020; Csordas et al., 2018; Soh et al., 2008; Wilms and Kleinebudde, 2020). The applied experimental designs can be assigned as process designs (Eriksson et al., 1998).

As indicated above, the quality of the ribbons and resutling products is also influenced by the applied materials and their physico-chemical attributes. Both, brittle and plastic materials are typically necessary to produce ribbons with good quality. Plastically deformable components thereby form new bonds under pressure by irreversibly deforming after exceeding the yield point, creating new contact surfaces and closer distances for new interparticle interactions, leading to mechanical interlocking. Microcrystalline cellulose (MCC) is frequently used as binder, but also hydroxypropyl cellulose grades or polyvinylpyrrolidones (Mangal et al., 2016). Such plastically deformable components are characterized by the formation of hard and mechanically resistant compacts during compaction. The relative sensitivity to specific compaction force (SSC) is for MCC thereby described by an exponential function, which accounts for a disctinct loss in tabletability (Janssen et al., 2022). Of particular interest for this work are MCC and silicified MCC (SMCC). SMCC is a type of MCC co-processed with highly dispersed silicon dioxide. The fine silicon dioxide particles are immobilized and evenly distributed on the surface of the MCC, thereby multiplying the specific surface area, improving the flow behavior and increasing the compactability (Alfa et al., 2006; van Veen et al., 2005).

Brittle materials, such as dicalcium phosphate (DCP), lactose, or mannitol are typically used as fillers (Janssen et al., 2022; Lück et al., 2022; Wagner et al., 2013; Yu et al., 2013). Such materials fragment into smaller particles under pressure, increasing the specific surface area for the formation of new interparticle interactions. Applying materials with less adequate binding properties for RC/DG can increase the residual fines, which would, in turn, negatively impact the flowability of the granules (Gamble et al., 2010). However, such materials are less impacted by the work hardening phenomenon compared to plastically deformable materials, as presented for two different lactose types by (Janssen et al., 2022). Finding the right formulation for roller compaction processes is thus also essential. Others than process designs, mixture designs help to assess product qualities based on changing mixture compositions and indicate to what extent the changes will affect the process-related properties of the mixture (Anderson-Cook et al., 2004; Snee, 1979).

The present study describes an iterative approach towards a ternary mixture design preceeded by a process design step. This is in contrast to the typical procedure in the industry, where usually the effect of process variations on the performance of a given formulation is evaluated as part of establishing the design space under Quality by Design rules (European Medicines Agency, 2017). Here, however, the aim was to identify suitable process conditions first to subsequently investigate the effect of substantial formulation changes. Specifically, the mixing ratios of the plastically deformable binders MCC and SMCC, as well as the brittle binder DCP, were systematically varied in a series of experiments. The resulting RC/DG granules were examined for their flow behavior, particle size distributions, bulk densities, and re-compressibility in a tableting step following roller compaction.

## 2 Materials and methods

### 2.1 Materials

Three materials were used for RC/DG studies: Microcrystalline cellulose (MCC, VIVAPUR® 101, JRS Pharma, predominantly plastic-deforming material), silicified microcrystalline cellulose (SMCC, PROSOLV® SMCC 50, JRS Pharma, predominantly plastic-deforming material), and dicalcium phosphate (DCP, Emcompress® Anhydrous Powder, JRS Pharma, brittle-deforming). Due to the dependency of DG/RC granule properties on the particle size of plastically deformable binders, the raw materials MCC and SMCC were selected with comparable particle sizes. Particle size distributions of the raw materials can be found in the supporting material (Fig. S1).

### 2.2 Experimental plan for the process design RC/DG studies

Prior to RC/DG, the raw material blends were mixed for 20 min with an angular frequency ω of 11 [min^−1^] in a V-mixer (120 L, JRS). Binary mixtures of MCC:DCP (Exp.1-8*a*) and SMCC:DCP (Exp.1-8*b*), were blended in a ratio of (3:1) per mixture. RC/DG was performed with a compactor (Walzenpresse WP 50 N/75, Alexanderwerk), equipped with press rolls (Cavex CHUA 99, Flender) and a feeding screw (H4V41, Heynau Gears Production Service). Compaction took place with a variable gap using a 7.5 cm broad, axially profiled roller. The uncompacted fine grains were discarded, whereas the slugs were coarsely crushed before granulating over an oscillating sieve with an 800 μm screen. The hopper agitator was kept constantly at level 5, whereas the feeding screw speed N_S_ [min^−1^], the roll speed N_R_ [min^−1^] and the specific compaction force SCF [kN/cm] were varied as part of the experimental design. For the purpose of this study, a non-automated compactor was selected in order to enable manual setting of all key variables. The screw-to-roll speed ratio ϑ (Table 1) was calculated as (N_S_/N_R_). The experiments followed a 2^3^ full factorial design (Table 1) for each of the two mixtures. Randomization of the experiments was generated by Minitab statistical software (21.4.0). In the manuscript, these trials are referred to as ‘Experiments’ (‘Exp.’).

**Table 1:** Factors and levels for roll compaction/dry granulation process design studies of (a) microcrystalline cellulose:dicalcium phosphate (3:1) and (b) silicified microcrystalline cellulose:dicalcium phosphate (3:1); The screw-to-roll speed ratio ϑ was calculated as (N_S_/N_R_) with N_S_ = screw speed [min^−1^] and N_R_ = roll speed [min^−1^].

Experiments (a) and (b)	Specific compaction force (SCF) [kN/cm]	Roll speed [min^−1^]	Screw speed [min^−1^]	Screw-to-roll speed ratio ϑ []
1	4.92	8	14	1.75
2	11.48	8	14	1.75
3	4.92	16	14	0.88
4	11.48	16	14	0.88
5	4.92	8	67	8.4
6	11.48	8	67	8.4
7	4.92	16	67	4.2
8	11.48	16	67	4.2

### 2.3 Experimental plan for the mixture design RC/DG studies

Prior to RC/DG, the raw material blends were mixed for 15 min with an angular frequency ω of 24 min^−1^ in a cube mixer (14 L, JRS). The cube mixer was chosen for these experiments due to the lower volume of the investigated blends. The raw materials were mixed in proportions following Table 2. The process parameters during roller compaction were kept constant across the mixture design experiments to attribute changes in the target size to variations in the mixture proportions (Snee, 1979) and to allow uncontrolled factors to uniformly affect the respective blocks.

**Table 2:** Mixture experimental design with the standard sequence of the experiments, block assignments, randomized sequence of experiments, and mixture proportions of microcrystalline cellulose (MCC), silicified microcrystalline cellulose (SMCC), and dicalcium phosphate (DCP).

Standard	Block	Run	MCC [%]	SMCC [%]	DCP [%]
4	1	1	67	33	0
13	1	2	16.5	16.5	67
10	1	3	0	33	67
14	1	4	100	0	0
15	1	5	0	100	0
5	2	6	67	0	33
12	2	7	16.5	67	16.5
9	2	8	0	67	33
3	2	9	0	0	100
16	2	10	0	0	100
11	2	11	67	16.5	16.5
6	3	12	33	67	0.0
8	3	13	33	0	67
17	3	14	33	33	33
2	3	15	0	100	0
7	3	16	33	33	33
1	3	17	100	0	0

The experimental design we followed is divided into 3 blocks (Table 2). Analysis of the products (Section 2.5) was carried out at same day for the granules of each block. Thereby, inconsistent influence on the experimental results of the analyzed target values within each block, e.g. by ensuring nearly identical environmental conditions, storage and transportation conditions for the granules of each block, were avoided. Once the experiments had been divided into their blocks, randomization of the experimental arrangement were performed within the blocks (Cornell, 2002; Siebertz et al., 2017). In the manuscript, these trials are referred to as ‘Runs’.

### 2.4 Tableting

Tableting of flat face tablets (13 mm in diameter) was performed with an instrumented tablet press (Pressima, IMA KILIAN). Sodium stearyl fumarate (PRUV®, JRS Pharma) was added as lubricant (1 %) to the RC/DG granules (resulting from Exp.1-8*a* and Exp.1-8*b*, Table 1) and mixed for 3 min in a cube mixer (AR 403, ERWEKA) at ω= 24 cm^−1^. RC/DG granules, and for comparison also the unprocessed materials as physical mixtures (MCC:DCP=PM*a*, SMCC:DCP=PM*b*), were tableted with different compression forces in the range between 2 and 10 kN during the process design experiments (Table 1) and between 2 and 15 kN for the experiments of the mixture design experiments (Table 2). Results are compared based on tablets produced at compression forces of 10 kN.

### 2.5 Analysis of particles and tablets

#### 2.5.1 Particle size evaluation of raw materials and granules

Raw materials and granules were analyzed over 60 s with a laser diffractometer (LS 13320, Beckman-Coulter) with the dry module Tornado 3 Powder System (AL02007), which swirles the powder sample in a turbulent airstream. A background measurement was carried out prior each measurement. The volumetric particle size distribution was determined according to Fraunhofer.

The data of a classified particle size distribution were converted into a continuous density distribution using Python (3.12.0) through cubic spline interpolation. This approach allows for the interpolation of the proportions of arbitrarily defined particle size fractions independently of the given particle size classes by integrating the continuous distribution density (Plato, 2021). Spline interpolation is a mathematical method of polynomial interpolation, in which a larger number of supporting points (in this case: value pairs consisting of particle size in the form of the class midpoint of a histogram and the relative frequency) are interpolated piecewise by a polynomial of n-th degree while maintaining predefined mathematical boundary conditions (Stieß, 2008). Following this approach, the proportion of the particle size fraction of the produced granules was determined, where the particles are larger than the d_90_ of the physical mixture (PM) before compaction. This analysis enables the assessment of the degree of agglomeration or the extent of the re-disintegration of the granules into their primary particles after granulation. In this study, this fraction is referred to as coarse faction. The fraction of particles smaller than the d_90_ of the physical mixture (PM) before compaction is defined as fines.

#### 2.5.2 Flow properties and densities of the granules

The powder flowability was measured as Angle of Repose according to method 2.9.36. of the European Pharmacopoeia (07/2024:20936, ‘Powder flow’), using a JRS-built apparatus (Fig. S2) with a line laser and digital calipers for contactless and precise determination of the height of the powder cone. Bulk and tapped density were determined following method 2.9.34. of the European Pharmacopoeia (04/2019:20934, ‘Bulk density and tapped density of powders’), particularly following ‘Method 1’ for both densities.

#### 2.5.3 Manufacturability and resistance to crushing of tablets

We determined the resistance of all tablets to crushing following method 2.9.8. of the European Pharmacopoeia (01/2008:20908, ‘Resistance to crushing of tablets’). As stated in the monograph, the results are expressed as breaking forces in [N]. This parameter was evaluated in correlation to the tableting compression force to yield the manufacturability according to the USP (United States Pharmacopeia, 2024).

### 2.6 Data analysis

#### 2.6.1 Process designs

Data of the process design experiments were evaluated with Minitab statistical software (21.4.0) and Python (3.12.0). The randomized 2^3^ full factorial design was generated by Minitab, the measured data evaluated with Python by polynomial interpolation. The effects of the individual factors were therefore calculated with $E_{i}=x_{\max,i}-x_{\min,i}$ where $x_{\max,i}$ is the mean of all experiments with factor i set to its highest level and $x_{\min,i}$ is the mean of all experiments with factor i set to its lowest level. Interactions were calculated as the difference between the mean response of all experiments where both factors were simultaneously set to their highest or lowest levels and the mean response of experiments where the two factors were set oppositely: $W_{i,j}=x_{++/--}-x_{+-/-+}$. Furthermore, Lagrange polynomial interpolation was applied to obtain a multivariate estimation function for the quality characteristics and to generate predictions within the chosen factor levels based on interpolation (Eq. (1)).

(1) $$\hat{y}\left(\mathrm{SCF},\omega ,N_{S}\right)=c_{\mathrm{SCF}}\mathrm{SCF}+c_{\omega }\omega +c_{N_{S}}N_{S}+c_{\mathrm{SCF\omega}}\mathrm{SCF\omega}+c_{\mathrm{SCF}N_{S}}\mathrm{SCF}N_{S}+c_{\omega N_{S}}\omega N_{S}+c_{\mathrm{SCF\omega}N_{S}}\mathrm{SCF\omega}N_{S}+k$$

The selected polynomial includes three different variables (SCF, angular frequency ω, and screw speed N_S_) representing the values of the factors. Each factor appears in a separate term with an associated coefficient that accounts for its effect. Additionally, three extra terms account for the two-factor interactions, one term represents the three-factor interaction, and one term serves as a model constant. By holding one variable constant (Eq. (2)), the setting of one factor required to achieve a given response $\hat{y}$ can be expressed as a function of another factor. Here, exemplarily, the SCF is represented as a function of the screw speed N_S_, which is also referred to as a contour line:

(2) $${\mathrm{SCF}}_{\left(N_{S}\right)}=\frac{\hat{y}-c_{\omega }\omega -c_{N_{S}}N_{S}-c_{N_{S}\omega }N_{S}\omega -k}{c_{\mathrm{SCF}}+c_{\mathrm{SCF\omega}}\omega +c_{\mathrm{SCF}N_{S}}N_{S}+c_{\mathrm{SCF\omega}N_{S}}N_{S}\omega }\left[\mathrm{kN}/\mathrm{cm}\right]$$

Since SCF and N_S_ can be continuously adjusted on the compactor, while the roller speed (N_R_) can only be varied at two levels, two contour plots can be generated for each quality characteristic based on the polynomial equations.

#### 2.6.2 Mixture designs

The mixture design was generated with Stat-Ease360, the measured data evaluated by multiple linear regression. Model equations for mixture design experiments can be generated using various mathematical methods, including multiple linear regression, where mixture proportions serve as independent variables to estimate the response. Scheffé model equations are, however, commonly used in this context (Piepel, 2014), which is why we developed Scheffé mixture models (Cornell, 2002) with Python (3.12.0). The most suitable Scheffé mixture model to predict the measured data was selected by several statistical criteria, including analysis of variance (ANOVA), stepwise backwards elimination starting from the special cubic Scheffé polynomial (Eq. (3)) as a saturated model.

(3) $$\hat{y}=\sum_{i=1}^{q}\beta_{i}x_{i}+\sum_{i=1}^{q-1}\sum_{j=i+1}^{q}\beta_{\mathrm{ij}}x_{i}x_{j}+\sum_{i=1}^{q-2}\sum_{j=i+1}^{q-1}\sum_{k=i+2}^{q}\beta_{\mathrm{ijk}}x_{i}x_{j}x_{k}$$

hereby, $\hat{y}$ is the predicted target value, x the variables (the fraction of components i,j,k), q is the amount of variables, and β are the calculated coefficients. When the experimental design was divided into b blocks, b−1 terms were added to the Scheffé polynomial (Eq. (4)).

(4) $$\hat{y}=\sum_{i=1}^{q}\beta_{i}x_{i}+\sum_{i=1}^{q-1}\sum_{j=i+1}^{q}\beta_{\mathrm{ij}}x_{i}x_{j}+\sum_{i=1}^{q-2}\sum_{j=i+1}^{q-1}\sum_{k=i+2}^{q}\beta_{\mathrm{ijk}}x_{i}x_{j}x_{k}+\sum_{i=1}^{b-1}\beta_{i,\mathrm{Block}}b_{i}$$

Each of these terms was associated with the independent indicator variable $b_{i}$ and the corresponding coefficient $\beta_{i,\mathrm{Block}}$.

We began the process of the stepwise backwards elimination with a full model that included all potential predictor variables of the response and their interactions. Based on ANOVA results, terms were progressively removed, if their contribution to explaining the variability of the measured response was minimal. First, the interaction term with the lowest adjusted sum of squares (SSR) was therefore eliminated if it was not significantly different from the mean square for regression (MSR) based on the F-test (*p* > 0.15, ‘Alpha-To-Remove’). A new regression model was then generated, and the process repeated until no further interaction terms met the criteria for elimination.

Since the linear effects of the mixture components form the basic model equations in this study and the mixture components were not independently variable, sequential SSR (Eq. (5)) were applied.

(5) $$\mathrm{SSR}\left(x_{\mathrm{SMCC}},x_{\mathrm{MCC}}|b_{1},b_{2}\right)=\mathrm{SSR}\left(x_{\mathrm{SMCC}},x_{\mathrm{MCC}},b_{1},b_{2}\right)-\mathrm{SSR}\left(b_{1},b_{2}\right)$$

These depend on the order in which predictor variables are added to the model and calculate the changes in SSR and MSR accordingly. The two categorical variables for the blocks b, which serve to account for uncontrolled factors, were first added to the model. The model was then extended with all linear effects of the mixture components, with the sequential sums of squares of the variance explained by the regression model.

If the modeling of the linear effects of the mixture components was eliminated at the end of the factor elimination process, the entire model was discarded. To make this verifiable, the following F-value is calculated (Eq. (6)).

(6) $$F_{\left(∆p,N-p,\alpha \right)}=\frac{\frac{\mathrm{SSR}\left(x_{p}|x_{i},\ldots ,x_{p-1}\right)}{∆{\mathrm{df}}_{\varepsilon }}}{\mathrm{MSE}}$$

The ANOVA tables generated in this study are structured according to Table 3. SSE is thereby the sum of squares error, also known as the residual sum of squares (RSS), is a measure of the total deviation of observed values from the predicted values in a model. Based on this parameter, the total sum of squares (SST), which measures the total variation in the observed data and serves as a baseline for comparing model performance, and the SSR_b_ (Table 3), the coefficient of determination (R^2^) was calculated (Eq. (7)):

(7) $$R^{2}=1-\frac{\mathrm{SSE}}{\mathrm{SST}-{\mathrm{SSR}}_{b}}$$

**Table 3:** Summarized terms and equations applied within the ANOVA. ${\hat{y}}_{i}$ = the predicted value of the estimation function, ${\bar{y}}_{i}$ = the arithmetic mean of analyzed data, *b* = number of blocks, *p* = number of predictors in the regression, *q* = number of parameters.

Source of variation	Degrees of freedom	Sum of squares (R = due to regression, E = due to errors, T = total)	Mean square (R = of regression, E = Error)
Blocks	${\mathrm{df}}_{\mathrm{block}}=b-1$	${\mathrm{SSR}}_{b}=\sum {\left({\hat{y}}_{i}-\bar{y}\right)}^{2}$	${\mathrm{MSR}}_{b}=\frac{\mathrm{SSR}\left(b_{1},b_{2}\right)}{b-1}$
Regression model	${\mathrm{df}}_{\mathrm{regression}}=p-1$	$\mathrm{SSR}=\sum_{i}^{N}{\left({\hat{y}}_{i}-\bar{y}\right)}^{2}$	$\mathrm{MSR}=\frac{\mathrm{SSR}}{p-1}$
Linear mixture	∆df=q−1	$\mathrm{SSR}\left(x_{\mathrm{SMCC}},x_{\mathrm{MCC}}|b_{1},b_{2}\right)$	$\mathrm{MSR}=\frac{\mathrm{SSR}\left(x_{\mathrm{SMCC}},x_{\mathrm{MCC}}|b_{1},b_{2}\right)}{q-1}$
Interactions	∆df=1	$\mathrm{SSR}\left(x_{p}|x_{i},\ldots ,x_{p-1}\right)$	$\mathrm{MSR}=\frac{\mathrm{SSR}\left(x_{p}|x_{i},\ldots ,x_{p-1}\right)}{1}$
Residuals	${\mathrm{df}}_{\varepsilon }=N-1-{\mathrm{df}}_{\mathrm{regression}}-{\mathrm{df}}_{\mathrm{block}}$	$\mathrm{SSE}=\sum_{i=1}^{N}{\left(y_{i}-{\hat{y}}_{i}\right)}^{2}$	$\mathrm{MSE}=\frac{\mathrm{SSE}}{N-p-b}$
Total	${\mathrm{df}}_{\varepsilon }=N-1={\mathrm{df}}_{\varepsilon }+{\mathrm{df}}_{\mathrm{regression}}+{\mathrm{df}}_{\mathrm{block}}$	$\mathrm{SST}=\sum_{i=1}^{N}{\left(y_{i}-\bar{y}\right)}^{2}$	–

To evaluate and compare different models the adjusted coefficient of determination $R_{\mathrm{adj}.}^{2}$ (Eq. (8)) and the predicted coefficient of determination $R_{\mathrm{pred}.}^{2}$ (Eq. (9)) were calculated:

(8) $$R_{\mathrm{adj}.}^{2}=1-\left(\frac{\mathrm{SSE}}{\mathrm{SST}-{\mathrm{SSR}}_{b}}*\frac{N-1}{N-p}\right)$$

(9) $$R_{\mathrm{pred}.}^{2}=1-\frac{\sum_{i=1}^{n}{\left(y_{i}-{\hat{y}}_{i\left(i\right)}\right)}^{2}}{\mathrm{SST}}$$

$R_{\mathrm{adj}.}^{2}$ thereby allows comparing different models without having a tendency to prefer models with a higher amount of predictor variables. $R_{\mathrm{pred}.}^{2}$ allows for the cross validation (based on the leave-one-out approach) of different models and thereby for comparing their ability to predict new data that was not included in the previous data set for the regression. $R_{\mathrm{pred}.}^{2}$ decreases with the addition of unsuited predictors for the forecast of new data and therefore serves as an indication for overfitting. Ideally, a model should have high and similar values for both coefficients of determination.

The applied Python codes are provided as Supplementary Material (Code_S1).

## 3 Results and discussions

### 3.1 Results and discussions of the process design experiments

**Fig. 1:**
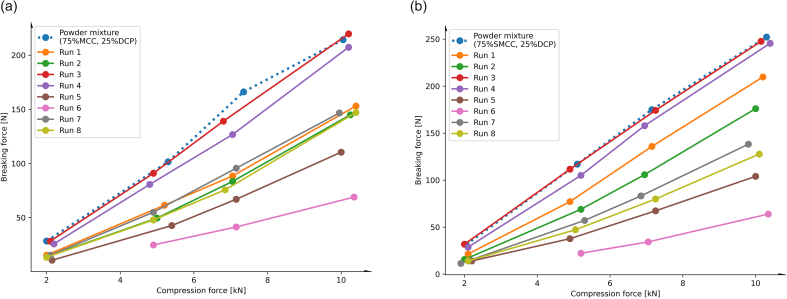
(a) Breaking forces of tablets based on granules and PM of MCC:DCP (3:1) and (b) breaking forces of tablets based on granules and PM of SMCC:DCP (3:1); tablets were compressed at compression forces between 2 and approx. 10 kN.

With all tableting experiments, the theory of loss in manufacturability due to initial RC/DG (Sun and Kleinebudde, 2016) was proven (Fig. 1). Thus, breaking forces of the tablets based on RC/DG material were almost without exception reduced, compared to those of the directly compressed physical mixture (PM). Further, breaking forces of the tablets were in the same order of magnitude for granules based on MCC and SMCC. This is in agreement with the literature, as both materials exhibit comparable compaction mechanisms (Bolhuis and Armstrong, 2006).

Overall, granules from Exp.3 and 4 (Table 1), conducted with high roll speed and low speed of the feeding screws (i.e. a low ϑ of 0.88) resulted in tablets with the highest breaking forces (Fig. 1a and b), comparable to the corresponding PMs. Comparison of the particle size distributions indicated that the respective RC/DG granules mainly matched those of the PM (Table 3; Fig. S1). This suggests that the process setting led to sub-feeding operating rates, i.e. the amount of provided powder by the screw feeder is too small and the material is scarcely compacted (Simon and Guigon, 2000). By contrast, RC/DG performed with the high level setting of the screw speed (N_S_ = 67 min^−1^) resulted in tablets with lower breaking forces and an increased coarse fraction. We defined the latter as particles larger than the d_90_ of the corresponding PMs (see Section 2.5.1), and identified that for Exp.5a-8a, 53–76 % of particles were larger than 128 μm (d_90_ of PMa), and that for Exp.5b-8b, 54–94 % of particles were larger than 143 μm (d_90_ of PMb). The coarse fraction results are reported in Table 4.

**Table 4:** Summary of the location parameters d_10_, d_50_, and d_90_ in [μm], the coarse fractions, defined as those particles > d_90_ of the corresponding physical mixtures (PM), and the slopes of the breaking force-to-compression force fits to compare the characteristics of the MCC:DCP (3:1) granules (indexed with ‘a’) with those of the SMCC:DCP (3:1) mixtures (indexed with ‘b’).

Material	Experiment	d_10_ [μm]	d_50_ [μm]	d_90_ [μm]	Coarse fraction (cfr) >d_90_ of PM [%]	Slope [N/kN]
MCC:DCP (3:1)	PMa	12	45	128	–	23.7
	Exp.1a	21	123	604	49	16.3
	Exp.2a	21	165	684	56	16
	Exp.3a	14	49	135	12	23.8
	Exp.4a	14	53	147	15	22.6
	Exp.5a	34	328	746	71	12.7
	Exp.6a	41	402	845	76	9.1
	Exp.7a	22	146	662	53	16.8
	Exp.8a	23	149	673	54	15.9
SMCC:DCP (3:1)	PMb	14	56	143	–	26.8
	Exp.1b	15	63	228	19	23.5
	Exp.2b	23	151	685	51	20.1
	Exp.3b	14	52	129	7	26.5
	Exp.4b	14	51	127	6	26.3
	Exp.5b	33	345	761	67	11.6
	Exp.6b	252	652	970	94	10
	Exp.7b	24	173	680	54	16
	Exp.8b	28	264	705	61	16.8

The biggest coarse fractions were obtained in Exp.5 and Exp.6 of both material mixtures, each performed with the highest screw-to-roll speed ratio ϑ of the process design experiments. This underlines some literature findings about the impact of the roll speed (N_R_). Since the blend is predominantely based on a plastically deformable material, precentage of fine material (in our case < d_90_) is decreased (Al-Asady et al., 2016). This effect is more pronounced with increasing SCF. The narrowest (and monomodal) particle size distribution was thus observed for process settings of Exp.6b, wherein a high ϑ was combined with high specific compaction force. Compared to tablets from Exp.6, tablets from Exp.5 exhibited good manufacturability (Fig. 1). While ϑ was the same between Exp.5 and 6, we assume the higher applied SCF in Exp.6 to have caused a reduced porosity of the ribbons (Olaleye et al., 2020). Such reduction would explain the decreased tensile strength of the resulting tablets, known as work-hardening (Sun and Kleinebudde, 2016).

Independent of the prior RC/DG setting, however, blends containing SMCC tended to produce tablets of somewhat higher strength (Fig. 1b). Though, in contrast to blends containing MCC, each RC/DG setting resulted for blends with SMCC in a slightly different manufacturability. Thereby, the manufacturability was more comparable for those granules, which were prepared with higher roll speeds (N_R_ = 16 min^−1^). This resulted in slopes of 16 [N/kN] for Exp.7b versus 16.8 [N/kN] for Exp.8b and 26.5 [N/kN] for Exp.3b versus 26.3 [N/kN] for Exp.4b (Table 4). RC/DG granules prepared with the low roll speed setting (N_R_ = 8 min^−1^) were more affected by the SFC, whereby a higher SFC resulted in a more pronounced loss in manufacturability (Table 4; 10 [N/kN] for Exp.6b versus 11.6 [kN/N] for Exp. 5b and 20.1 [N/kN] for Exp.2b versus 23.5 [N/kN] for Exp.1b).

**Fig. 2:**
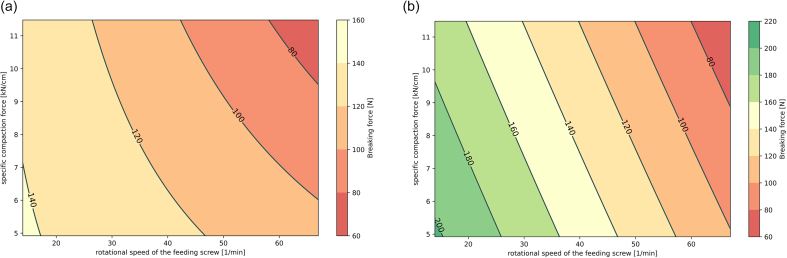
Contour plots of the breaking forces [N] of tablets (relating to a tableting compression force of 10 kN). RC/DG material of (a) MCC:DCP and (b) SMCC:DCP was produced at a constant roll speed (w = 8 min^−1^), varying specific compaction forces and screw speeds.

The related contour diagrams of the breaking forces of the tablets (Fig. 2a and b) display that at a constant roll speed of N_R_ = 8 min^−1^, the interaction of the two variable factors specific compaction force and screw speed had a more pronounced effect on breaking force in the SMCC test series. This supports what has been indicated already in Fig. 1 and Table 4, namely that formulations with MCC might be more robust in terms of changes to process settings, whereas formulations with SMCC enable higher final tablet strength at the same processing conditions.

**Fig. 3:**
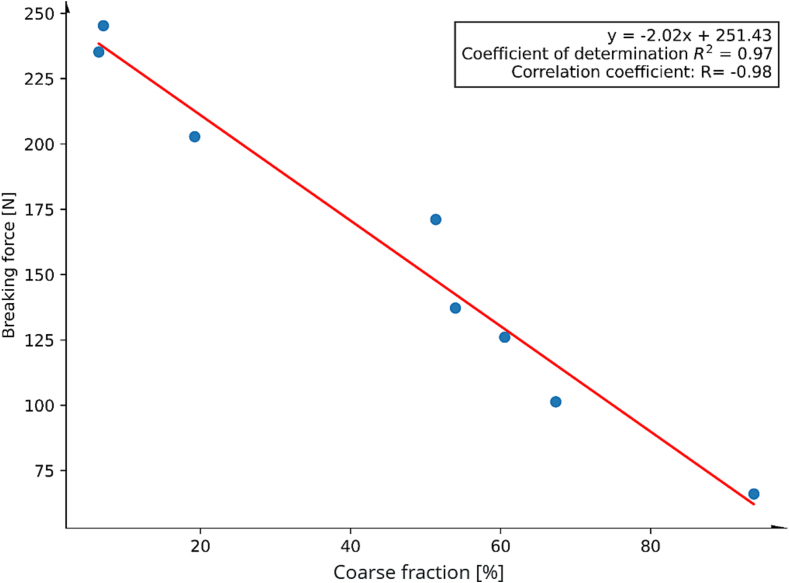
Correlation between the coarse fraction and the breaking force of the SMCC:DCP (3:1) tablets (weighing 500 mg, relating to a tableting compression force of 10 kN).

In agreement with literature (Sun and Himmelspach, 2006), we found a correlation between the breaking forces and the coarse fraction for both MCC:DCP and SMCC:DCP (shown in Fig. 3 for SMCC:DCP, for MCC:DCP please refer to Fig. S3).

**Fig. 4:**
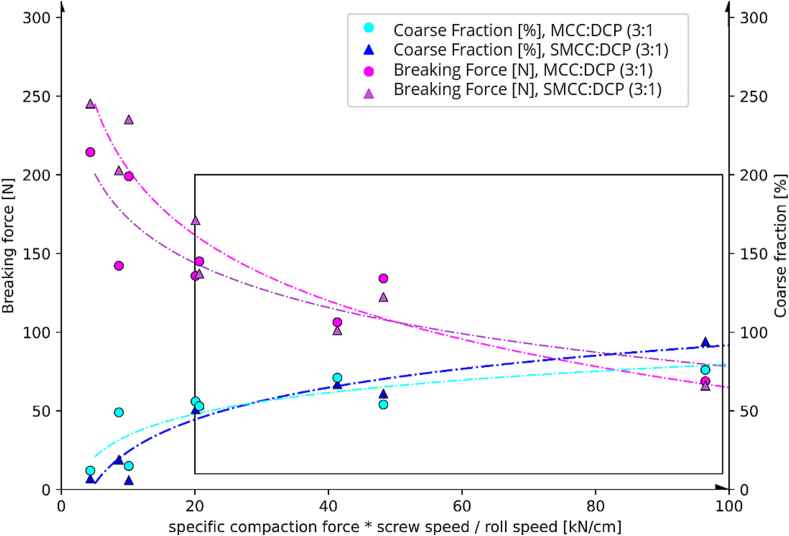
Interrelation of the coarse fraction and the breaking force of tablets (weighing 500 mg, relating to a tableting compression force of 10 kN) in combination against the specific compaction force multiplied with the screw-to-roll speed ratio ϑ [kN/cm].

Data shown in this study and throughout the literature indicate, that the intensity of the roller compaction grows with increasing screw speed, decreasing roller speed and increasing SCF. While screw and roller speed are commonly combined in the quotient ϑ, SCF is often considered in isolation. We found, however, that combining the three by multiplying the specific compaction force (SCF) with the screw-to-roll speed ratio ϑ lead to a meaningful new factor. Therefore, we further plotted the coarse fractions of the granules and the breaking forces of the related tablets against this factor. As per the numbers from Table 1, ‘SCF * ϑ’ factor, expressed in [kN/cm], ranged from 4.33 for Exp.3 to 96.43 for Exp.6 (for all numbers see Table S1). The obtained function (Fig. 4) fits the finding of (Janssen et al., 2022), that the hardness of RC/DG MCC tablets decreases with SCF.

In our experiments, we identified 20 (SCF * ϑ) [kN/cm] as the minimum setting to get granules with sufficient coarse particle fractions and ∼ 60 (SCF * ϑ) [kN/cm] as the maximum setting that allows for the production of sufficiently hard tablets (Fig. 4).

### 3.2 Results and discussions of the mixture design experiments

With the optimal processing range determined to be between 20 (SCF * ϑ) [kN/cm] and ∼ 60 (SCF * ϑ) [kN/cm], we selected a SCF of 8.2 [kN/cm], a screw speed of N_S_ = 50 cm^−1^, and a roller speed of N_S_ = 8 cm^−1^, resulting in a ‘SCF * ϑ’ factor of 51.25 [kN/cm]. The funnel agitator was set to level 5 and the roller compaction was carried out using axially profiled press rollers with a width of 7.5 cm and a variable gap.

Using these settings, we aimed to find the ‘best’ formulation based on the three investigated excipients based on Scheffé models (Cornell, 2002), following the approach of Snee (Snee, 1979). Therefore, the proportion of any component in the mixture must lie between 0 and 1 and the sum of all proportions was set to 1.

Since the measured values of the target variables considered in this study, such as flow behavior and re-compressibility, are significantly influenced by particle size (Sun and Himmelspach, 2006), the particle size distribution provides clues for interpreting the results. In order to assess the reproducibility of the results and to identify possible causes for differences in the results, the particle size distributions of the replications are particularly important and will be discussed with regard to material in the following.

#### 3.2.1 Particle size distributions and further target variables of replicates

Initially, the particle size distributions of the replicates were compared to assess possible fluctuations in the target variables. In this context, RC/DG of 100 % DCP in Run 9 and 10 (Fig. S4) repeatedly resulted in a reduction in particle size compared to the uncompacted raw material (Fig. S1). The breaking force of the resulting tablets (relating to a compression force of 10 kN) was comparably low with 12 N, and 15 N, respectively (Table S2).

RC/DG of 100 % MCC in Run 4 and 17 reproducibly resulted in significant particle size enlargement relative to its uncompacted powder (see Figs. S1 and S4). Resulting tablets provided breaking force of 80 N, and 67 N, respectively. The particle size distributions of the dry granulates of pure SMCC in Run 5 and 15 (Fig. S4) exhibited similar results to those obtained with pure MCC. However, resulting breaking forces of the tablets were comparably higher with 132 N, and 120 N, respectively.

Experiments with ternary mixtures (Run 14 and 16) still reproducibly resulted in significant particle size enlargement compared to the PMs. The tablets' breaking forces, however, dropped to 73 N for tablets of both runs (Table S2).

#### 3.2.2 Particle size distributions and further target variables of designed mixtures

**Fig. 5:**
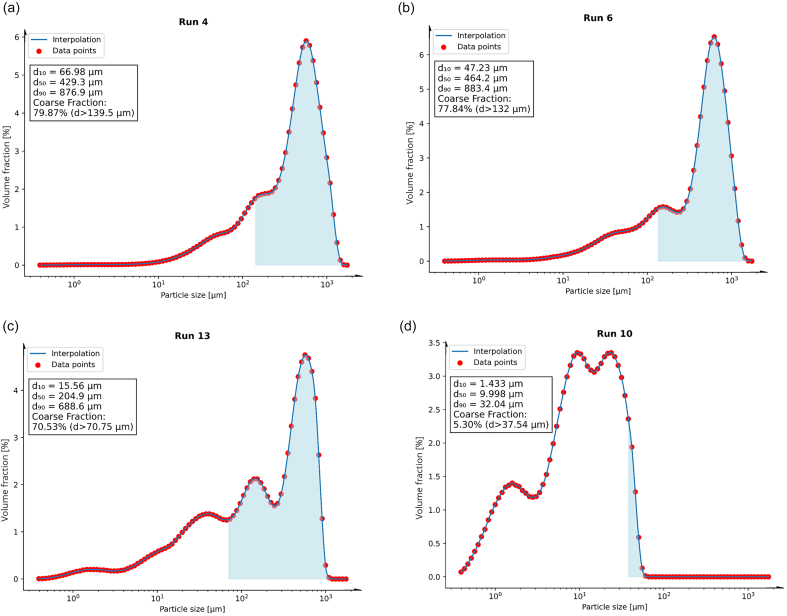
PSD of granules with decreasing proportion of MCC from (a) to (d), whereby (a) displays the results of granules based on 100 % MCC with 0 % DCP; (b) displays the results of granules based on 67 % MCC; (c) displays the results of granules based on 33 % MCC; (d) displays the results of granules based on 0 % MCC with 100 % DCP; areas in blue correspond to particle sizes > d_90_ of the related physical mixtures, defined as coarse fraction (concrete numbers are given in brackets). (For interpretation of the references to colour in this figure legend, the reader is referred to the web version of this article.)

The particle size distributions obtained for the blends according to the mixture design (Table 2) show that proportions of the brittle component DCP up to 33 %, tend to have a minor influence on the percentile values of blends with SMCC and MCC. This is shown by way of example for Run 4 (100 % MCC) and Run 6 (67 % MCC, 33 % DCP) compared to Run 13 (33 % MCC, 67 % DCP) (Fig. 5a vs. b and c), indicating a percolation threshold (Boersen et al., 2015; Leuenberger et al., 1987; Pérez Gago and Kleinebudde, 2017) of MCC in between 33 % and 67 %. The resulting breaking forces of the binary mixtures (Table S2) further supported this finding. For RC/DG granules, consisting mainly of DCP, as shown for Run 13 (33 % MCC, 67 % DCP, Fig. 5c) and Run 10 (100 % DCP, Fig. 5d), the fine particle fraction expectedly increased with the proportion of the brittle component, further the bulk and tapped density increased and the breaking force of the tablets decreased (see Table S2). Analogous results as presented here for MCC/DCP binary mixtures were obtained for mixtures including SMCC (see Fig. S5).

**Fig. 6:**
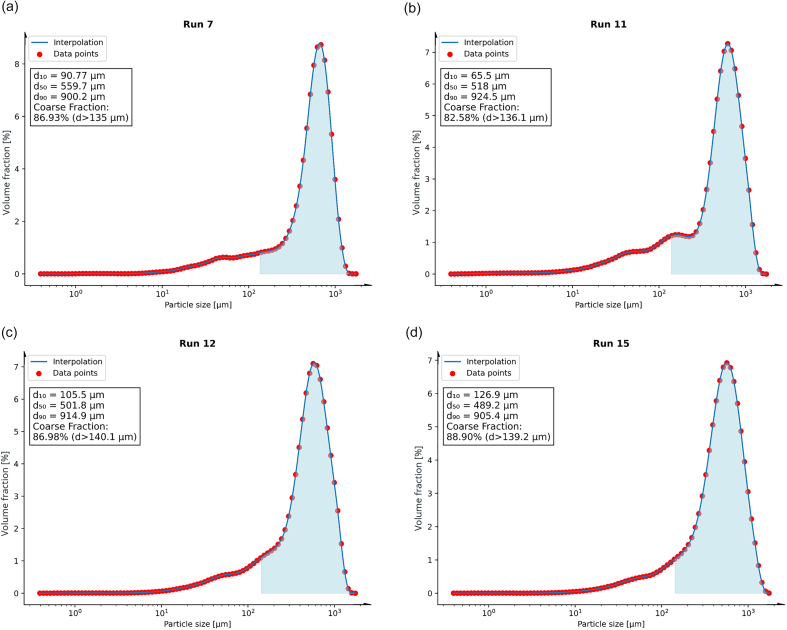
PSD of granules with the smallest amount of fines and almost monomodal distributions: Run 7 (67 % SMCC, 16.5 % MCC, 16.5 % DCP), Run 11 (67 % MCC, 16.5 % SMCC, 16.5 % DCP), Run 12 (67 % SMCC, 33 % MCC), and Run 15 (100 % SMCC). Areas in blue correspond to particle sizes > d_90_ of the related physical mixtures, defined as coarse fraction (concrete numbers are given in brackets). (For interpretation of the references to colour in this figure legend, the reader is referred to the web version of this article.)

In this work, the smallest amount of fines and almost monomodal distributions were observed whenever SMCC was part of the mixture (Fig. 6), particularly in Run 7 (67 % SMCC, 16.5 % MCC, 16.5 % DCP), Run 11 (67 % MCC, 16.5 % SMCC, 16.5 % DCP), Run 12 (67 % SMCC, 33 % MCC), and Run 15 (100 % SMCC).

#### 3.2.3 Description and interpretation of the contour plots

From a practical point of view, RC/DG is mainly performed to improve the bulk densities and flow properties of the starting materials while maintaining good compression properties, displayed by relevant breaking forces of resulting tablets. How these characteristics change in relation to different mixtures of SMCC, MCC, and DCP will be discussed in the following.

To evaluate the bulk density results, an ANOVA of the cubic model:

$$\hat{y}=0.46661918*x_{\mathrm{MCC}}+0.48058554*x_{\mathrm{SMCC}}+1.09447556*x_{\mathrm{DCP}}+0.13804332*x_{\mathrm{MCC}}*x_{\mathrm{SMCC}}-0.44953436*x_{\mathrm{MCC}}*x_{\mathrm{DCP}}-0.38663633*x_{\mathrm{SMCC}}*x_{\mathrm{DCP}}-0.51657653*x_{\mathrm{MCC}}*x_{\mathrm{SMCC}}*x_{\mathrm{DCP}}$$

gave results summarized in Table 5.

**Table 5:** ANOVA results of the special cubic model for bulk densities including F- and *p*-values.

Source of variation	Degrees of freedom	Sum of squares	Mean square	F	p
Blocks	2	0.1066	0.0533	–	–
Regression model	6	0.5786	0.0964	249.5979	0.0000
Linear mixture	2	0.5431	0.2715	716.9179	0.0000
SMCC*MCC	1	0.0013	0.0013	3.3920	0.1028
SMCC*DCP	1	0.0131	0.0131	34.6338	0.0004
MCP*DCP	1	0.0093	0.0093	24.6804	0.0011
SMCC*MCC*DCP	1	0.0004	0.0004	1.0452	0.3365
Residuals	8	0.0030	0.0004	–	–
Total	16	0.6882	–	–	–

Since the *p* value of the three-way interaction of SMCC*MCC*DCP was higher than the set Alpha-To-Remove (*p* = 0.3365 > *p* = 0.15, see Section 2.6.2), we followed a stepwise regression through backward elimination and calculated a new (quadratic) model without this factor:

$$\hat{y}=0.46794430*x_{\mathrm{MCC}}+0.48273134*x_{\mathrm{SMCC}}+1.095489314*x_{\mathrm{DCP}}+0.10548302*x_{\mathrm{MCC}}*x_{\mathrm{SMCC}}-0.47837523*x_{\mathrm{MCC}}*x_{\mathrm{DCP}}-0.42454787*x_{\mathrm{SMCC}}*x_{\mathrm{DCP}}$$

here, the two-way interaction of SMCC*MCC exceeded with *p* = 0.1554 the set ‘Alpha-To-Remove’, which is why we also analyzed results of the following, also quadratic model:

$$\hat{y}=0.476838*x_{\mathrm{MCC}}+0.49104464*x_{\mathrm{SMCC}}+1.09478867*x_{\mathrm{DCP}}-0.48045552*x_{\mathrm{MCC}}*x_{\mathrm{DCP}}-0.42021363*x_{\mathrm{SMCC}}*x_{\mathrm{DCP}}$$

The analysis of the coefficients of determination (Table 6) indicates that the special cubic, the full quadratic, and the quadratic model N°7, which was obtained by the backward elimination regression, achieved the best fits (Table 6, N°7–9) with minimal differences between R^2^_adj._ and R^2^_pred._ and low SSE values. Since adding parameters to model N°7 reduces R^2^_pred._ and due to the small difference from the full quadratic model (N°8), the quadratic model N°7, excluding two-way interactions of MCC and SMCC, was chosen as the final model.

**Table 6:** Summarized sum of squares due to error (SSE), coefficients of determination (R^2^, R^2^_adj._ and R^2^_pred._) for obtained models; model N° 1, 8, and 9 are full models, N° 2–4 are quadratic models containing one two-factor interaction, N° 5–7 are quadratic models containing two two-factor interactions.

Bulk density results model N°	Short description	SSE	R^2^	R^2^_adj._	R^2^_pred._
1	Linear model	0.0385	0.9338	0.9227	0.8702
2	+MCC*SMCC	0.0378	0.9351	0.9174	0.8437
3	+MCC*DCP	0.0187	0.9679	0.9592	0.9253
4	+SMCC*DCP	0.0217	0.9627	0.9525	0.9082
5	+MCC*SMCC+MCC*DCP	0.0180	0.9690	0.9567	0.9110
6	+MCC*SMCC+SMCC*DCP	0.0206	0.9645	0.9503	0.8937
7	+MCC*DCP + SMCC*DCP	0.0043	0.9925	0.9895	0.9751
8	Full quadratic model	0.0034	0.9941	0.9908	0.9749
9	Special cubic model	0.0030	0.9948	0.9909	0.9690

**Fig. 7:**
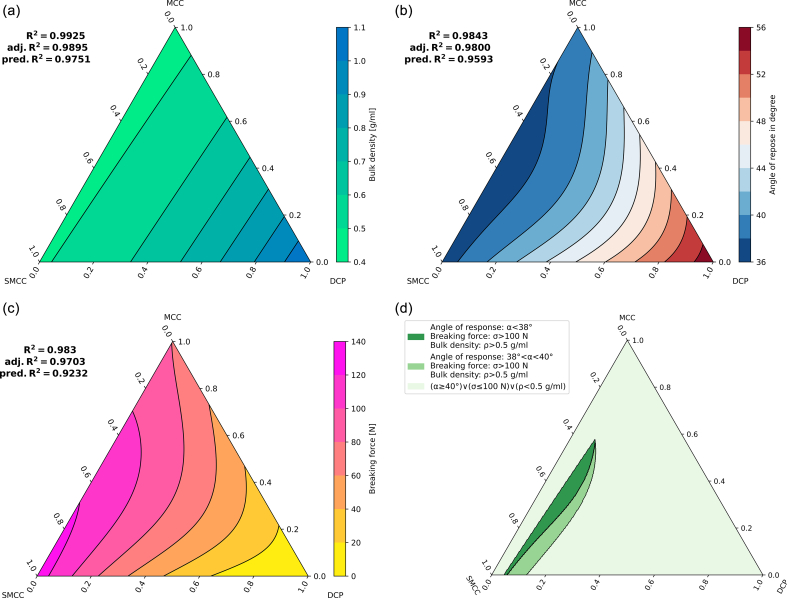
Contour plots of the mixture design models for (a) the bulk density, (b) the flow properties, (c) the breaking force, and (d) a superimposed mixture design model of combined target parameters bulk density, flow properties, and breaking force.

This mixture design model demonstrates on the one hand that the bulk density increases as the proportion of DCP increases (Fig. 7a). Granules made from pure MCC on the other hand had the lowest bulk density, which differed only slightly from that of SMCC. In sum, the more DCP was contained in a mixture with either MCC or SMCC, the more the bulk density increased. This is expressed in the contour triangle through a closer arrangement of the contour lines, the height values of which are graduated equidistantly in steps of 0.1 g/mL.

Following the same approach, we obtained for the angle of repose the following model with R^2^ = 0.9843, R^2^_adj._ = 0.9800 and R^2^_pred._ = 0.9593:

$$\hat{y}=38.1802098*x_{\mathrm{MCC}}+36.95620978*x_{\mathrm{SMCC}}+55.29636435*x_{\mathrm{DCP}}-77.93119874*x_{\mathrm{MCC}}*x_{\mathrm{SMCC}}*x_{\mathrm{DCP}}$$

The resulting contourplot (Fig. 7b) displayed a tendency towards lower slope angles and thus a higher flowability for dry granules with higher SMCC proportions, which is in agreement with the best flowability of the material produced in Run 5 (100 % SMCC, α = 36.96°). On the contrary, granules based on a high DCP content had the poorest flow properties. This is probably due to the high amount of fines of the raw material.

For the breaking forces of related tablets, which were compressed with 15 kN, the following model (with R^2^ = 0.9895, R^2^_adj._ = 0.9852 and R^2^_pred._ = 0.9665) was built:

$$\hat{y}=149.550608*x_{\mathrm{MCC}}+211.0482628*x_{\mathrm{SMCC}}-0.04932867131556254*x_{\mathrm{DCP}}-147.479339*x_{\mathrm{SMCC}}*x_{\mathrm{DCP}}+836.556338*x_{\mathrm{MCC}}*x_{\mathrm{SMCC}}*x_{\mathrm{DCP}}$$

The related contourplot (Fig. 7c) shows that tablets tend towards higher breaking forces the more SMCC is used instead of MCC as a dry binder in roller compaction. This might be of particular interest for the choice of the dry binder, as MCC is also known to be more sensitive towards the use of lubricants in tableting than SMCC (van Veen et al., 2005).

Following the suggestion of the Appendix 2C of the ICH Q8 (European Medicines Agency, 2017), a further model was built based on the three aforementioned important target parameters.

One resulting superimposed contour plot is shown in Fig. 7d. In our particular case, three areas of combined target values were set - the optimal combination was defined for granules resulting in an angle of repose of α < 38°, bulk densities of ρ > 0.5 g/mL and (tablet) breaking forces of σ > 100 N. With setting an angle of repose between 38° < α < 40°, the optimal mixture would allow for more DCP. Contour plots of the mixture design models for the fines, the d_50_ value, and the Hausner ratio can be found in Fig. S6.

## 4 Conclusion

Applying the selected 2^3^ full factorial design to binary mixtures of DCP and MCC / SMCC respectively, the effect of screw speed, roll(er) speed and specific compaction force on the PSD and recompactability of the produced RC/DG granules was evaluated. The resistance of tablets (towards crushing) made from RC/DG granules was found to be inversely proportional to the coarse fraction of the corresponding granules. Furthermore, we found that multiplying the specific compaction force with the screw-to-roll speed ratio ϑ led to a meaningful parameter, representing the intensity of the granulation step.

The subsequent mixture design was performed based on optimal process settings, namely a ‘SCF * ϑ’ factor of 51.25 [kN/cm] and allowed to investigate the impact of the three chosen excipients on the quality and manufacturability of RC/DG granules. Our findings indicate that using SMCC instead of or in mixtures with MCC leads to improved RC/DG granule properties. A superimposed mixture design model based on precise target values of the parameters bulk density, flow properties, and breaking force allowed identification of the best formulation.

## Chemical compounds studied in the article

Microcrystalline cellulose, silicified microcrystalline cellulose, dicalcium phosphate.

## CRediT authorship contribution statement

**Niclas Märkle:** Conceptualization, Data curation, Formal analysis, Investigation, Methodology, Software, Validation, Visualization, Writing – original draft. **Gernot Warnke:** Conceptualization, Methodology, Resources, Validation, Writing – original draft, Writing – review & editing. **Miriam Pein-Hackelbusch:** Conceptualization, Methodology, Supervision, Validation, Writing – original draft, Writing – review & editing.

## Declaration of competing interest

The authors declare the following financial interests/personal relationships which may be considered as potential competing interests:

Niclas Märkle reports financial support, article publishing charges, and equipment, drugs, or supplies were provided by JRS Pharma GmbH & Co KG. Gernot Warnke reports financial support was provided by JRS Pharma GmbH & Co KG. Gernot Warnke reports a relationship with JRS Pharma GmbH & Co KG that includes: employment. If there are other authors, they declare that they have no known competing financial interests or personal relationships that could have appeared to influence the work reported in this paper.

## Data Availability

Data and codes are shared as supplementary material.

## Appendix A Supplementary data

Supplementary material 1 Image 1

Supplementary material 2 Image 2

Supplementary material 3 Image 3

Supplementary material 4 Image 4

Supplementary material 5 Image 5

Supplementary material 6 Image 6

Supplementary material 7 Image 7
