# Supplementary material for: An iterative process and mixture design approach for dry granulated ternary blends of filler-binders
Source: Int J Pharm X. 2025 Apr 1;9:100331. doi: 10.1016/j.ijpx.2025.100331 (PMC12002786; doi:10.1016/j.ijpx.2025.100331)
Supplement: Supplementary file 1 — Supplementary material 1 [file mmc1.docx]

*Table S 1 Factors and levels for roll compaction/dry granulation process design studies of (a) microcrystalline cellulose:dicalcium phosphate (3:1) and (b) silicified microcrystalline cellulose:dicalcium phosphate (3:1); The screw-to-roll speed ratio ϑ was calculated as (N_S_/N_R_) with N_S_ = screw speed [min^-1^] and N_R_ = roll speed [min^-1­^].*

| **Experiments (a) and (b)** | **Specific Compaction Force (SCF) [kN/cm]** | **Roll Speed [min^-1^]** | **Screw Speed [min^-1^]** | **Screw-to-roll speed ratio ϑ [ ]** | **SCF * *ϑ***  **[kN/cm]** |
| --- | --- | --- | --- | --- | --- |
| 1 | 4.92 | 8 | 14 | 1.75 | 8.61 |
| 2 | 11.48 | 8 | 14 | 1.75 | 20.09 |
| 3 | 4.92 | 16 | 14 | 0.88 | 4.33 |
| 4 | 11.48 | 16 | 14 | 0.88 | 10.10 |
| 5 | 4.92 | 8 | 67 | 8.4 | 41.33 |
| 6 | 11.48 | 8 | 67 | 8.4 | 96.43 |
| 7 | 4.92 | 16 | 67 | 4.2 | 20.66 |
| 8 | 11.48 | 16 | 67 | 4.2 | 48.22 |

*Table S 2 Summarized material characteristics of the granules and tablets (*related to compression forces of 10 kN) produced during the mixture design experiments.*

| **Standard sequence** | **Block** | **Run** | **MCC** | **SMCC** | **DCP** | **Bulk density** | **Tapped density [mg/ L]** | **Hausner ratio** | **Slope height [mm]** | **Slope angle [°]** | **FLODEX [mm]** | **fine particle fraction [%]** | **d_50_** | **breaking force of tablets* [N]** |
| --- | --- | --- | --- | --- | --- | --- | --- | --- | --- | --- | --- | --- | --- | --- |
| N° | N° | N° | [%] | [%] | [%] | [mg L^-1^] | [mg L^-1^] | [ ] | [mm] | [°] | [mm] | [%] | [µm] | [N] |
| 4 | 1 | 1 | 67 | 33 | 0 | 0.488 | 0.610 | 1.25 | 22.5 | 37.8 | 14 | 21.9 | 409.7 | 110 |
| 13 | 1 | 2 | 16.5 | 16.5 | 67 | 0.789 | 1.031 | 1.31 | 35.2 | 50.5 | 24 | 31.6 | 176.4 | 21 |
| 10 | 1 | 3 | 0 | 33 | 67 | 0.792 | 1.020 | 1.29 | 34.6 | 50.0 | 22 | 30.4 | 213.7 | 22 |
| 14 | 1 | 4 | 100 | 0 | 0 | 0.438 | 0.559 | 1.28 | 23.2 | 38.7 | 16 | 20.1 | 429.3 | 80 |
| 15 | 1 | 5 | 0 | 100 | 0 | 0.453 | 0.575 | 1.27 | 21.8 | 37.0 | 14 | 21.2 | 466.5 | 132 |
| 5 | 2 | 6 | 67 | 0 | 33 | 0.615 | 0.758 | 1.23 | 26.5 | 42.4 | 16 | 22.2 | 464.2 | 65 |
| 12 | 2 | 7 | 16.5 | 67 | 16.5 | 0.534 | 0.645 | 1.21 | 22.8 | 38.2 | 12 | 13.1 | 559.7 | 113 |
| 9 | 2 | 8 | 0 | 67 | 33 | 0.602 | 0.763 | 1.27 | 26.0 | 41.8 | 16 | 18.8 | 467.7 | 69 |
| 3 | 2 | 9 | 0 | 0 | 100 | 1.103 | 1.538 | 1.39 | 39.7 | 53.8 | 26 | 91.0 | 13.1 | 12 |
| 16 | 2 | 10 | 0 | 0 | 100 | 1.110 | 1.563 | 1.41 | 40.1 | 54.1 | 26 | 94.6 | 10.0 | 15 |
| 11 | 2 | 11 | 67 | 16.5 | 16.5 | 0.516 | 0.658 | 1.27 | 23.6 | 39.2 | 14 | 17.4 | 518.0 | 92 |
| 6 | 3 | 12 | 33 | 67 | 0.0 | 0.517 | 0.617 | 1.19 | 22.4 | 37.7 | 12 | 13.0 | 501.8 | 100 |
| 8 | 3 | 13 | 33 | 0 | 67 | 0.761 | 1.020 | 1.34 | 34.1 | 49.6 | 22 | 29.8 | 204.9 | 23 |
| 17 | 3 | 14 | 33 | 33 | 33 | 0.599 | 0.758 | 1.26 | 24.1 | 39.8 | 14 | 29.1 | 363.2 | 73 |
| 2 | 3 | 15 | 0 | 100 | 0 | 0.501 | 0.595 | 1.19 | 22.7 | 38.0 | 12 | 11.1 | 489.2 | 120 |
| 7 | 3 | 16 | 33 | 33 | 33 | 0.582 | 0.752 | 1.29 | 24.6 | 40.3 | 14 | 24.9 | 398.3 | 73 |
| 1 | 3 | 17 | 100 | 0 | 0 | 0.479 | 0.595 | 1.24 | 23.6 | 39.2 | 16 | 17.1 | 446.7 | 67 |

| 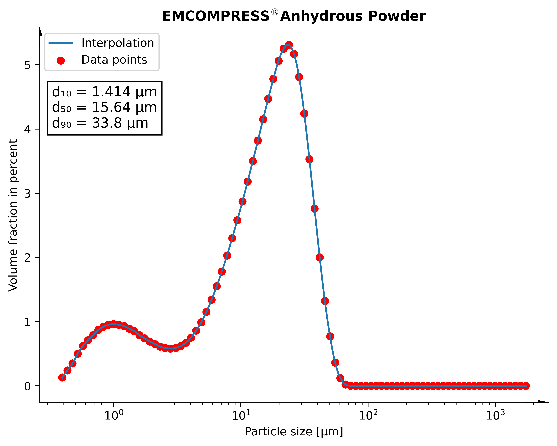 | 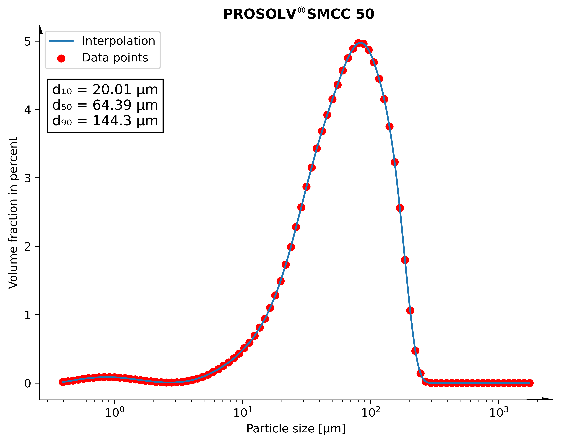 |
| --- | --- |
| 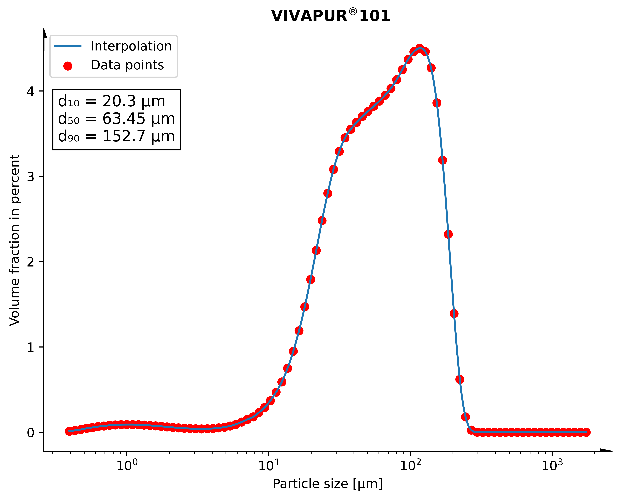 | 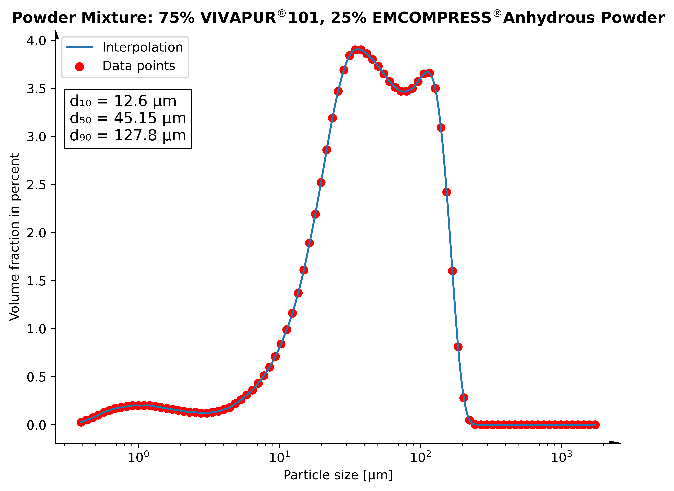 |
| 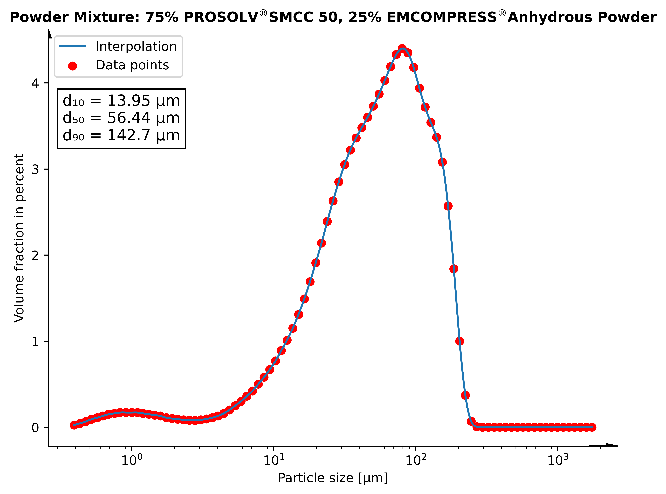 | 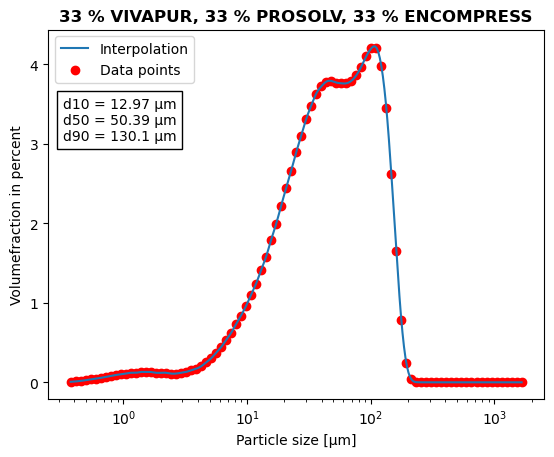 |

*Figure S 1 PSD of the raw materials MCC (VIVAPUR 101), SMCC (PROSOLV SMCC 50) and DCP (ENCOMPRESS Anhydrous Powder) and mixtures thereof.*


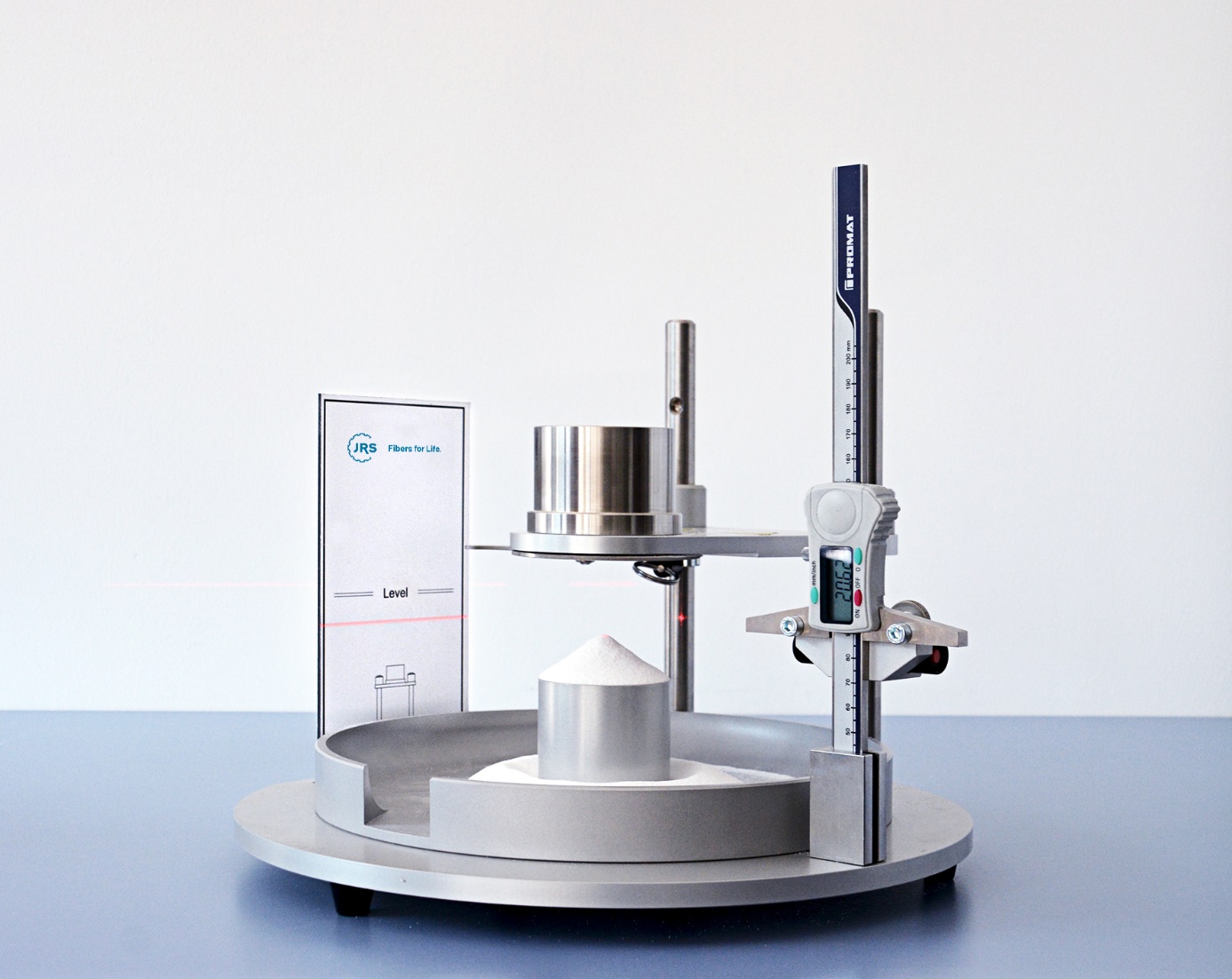


*Figure S 2 An apparatus to measure the Angle of Repose according to method 2.9.36. of the European Pharmacopoeia (07/2024:20936, ‘Powder Flow’) [built by JRS Pharma].*


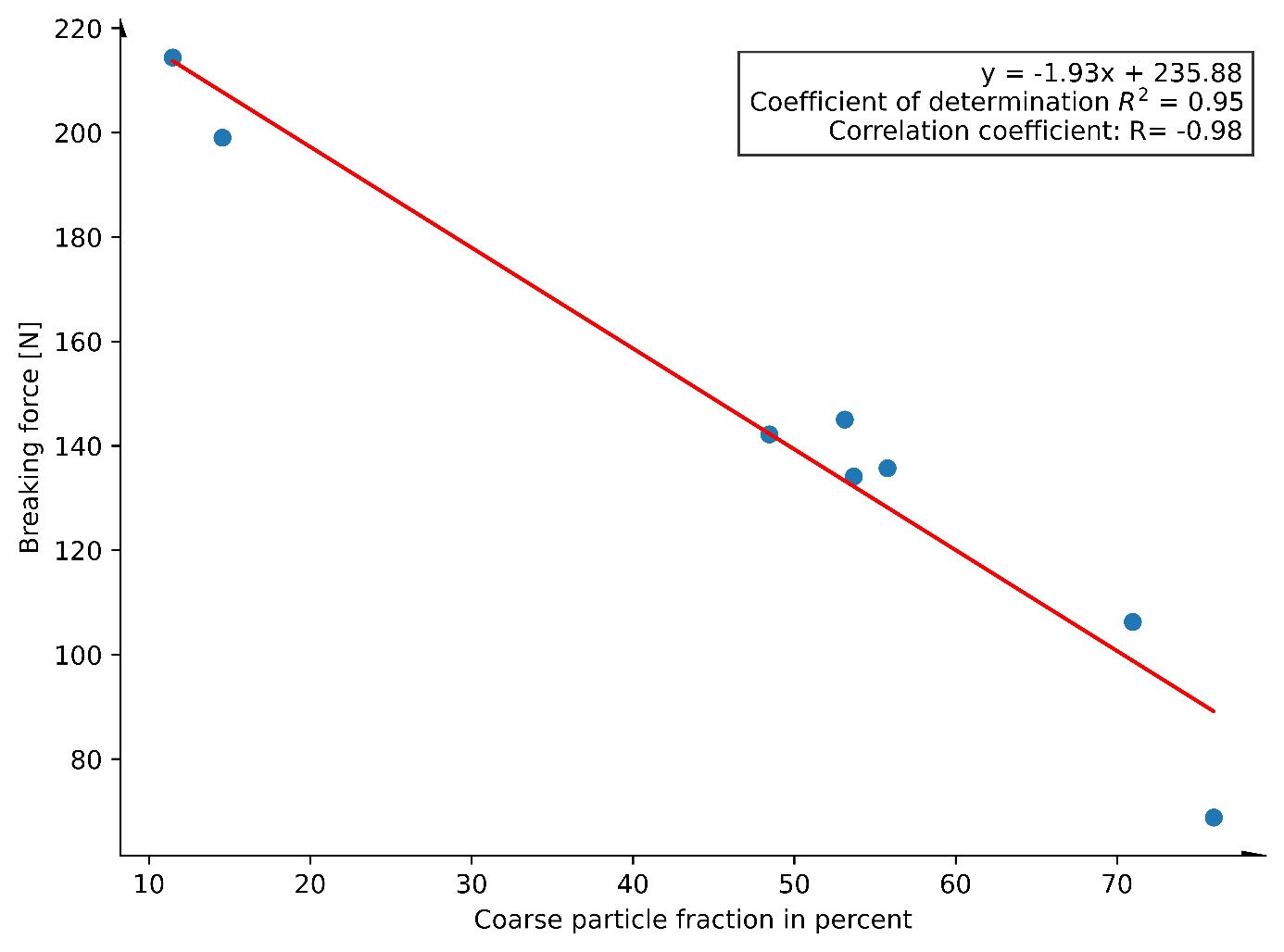


*Figure S 3 Correlation between the coarse fraction and the breaking force of the MCC:DCP (3:1) tablets (weighing 500 mg, relating to a tableting compression force of 10 kN).*

| 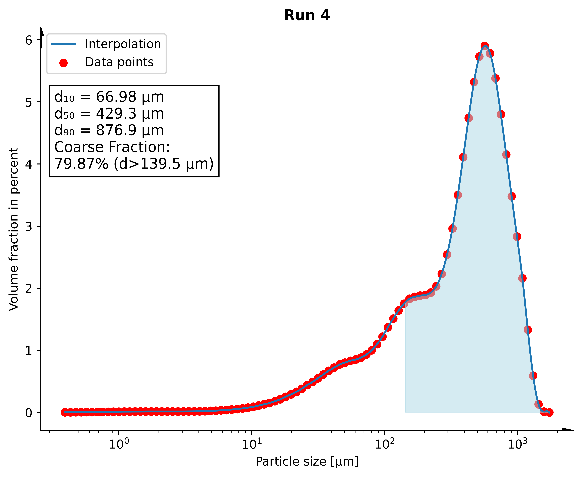 | 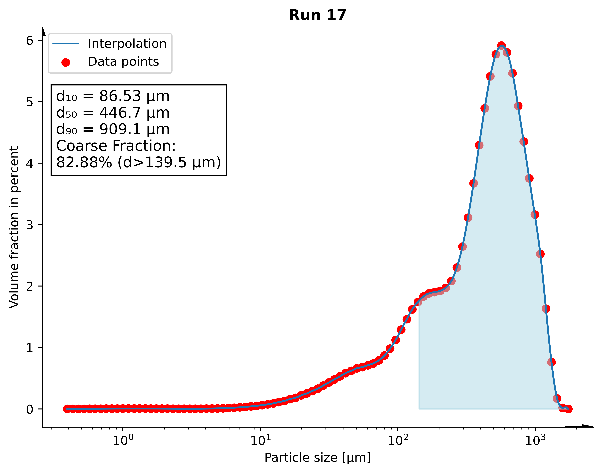 |
| --- | --- |
| 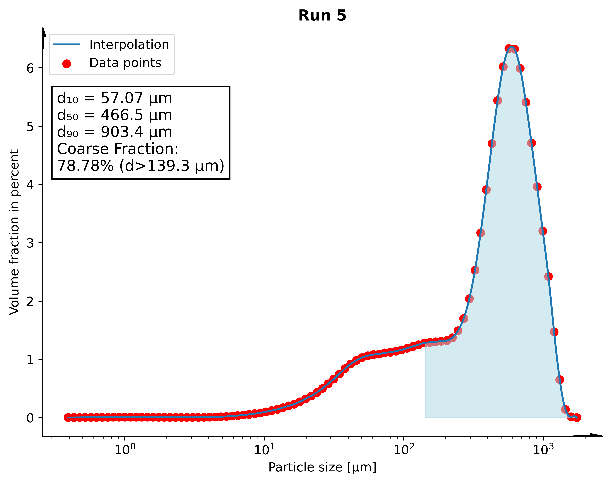 | 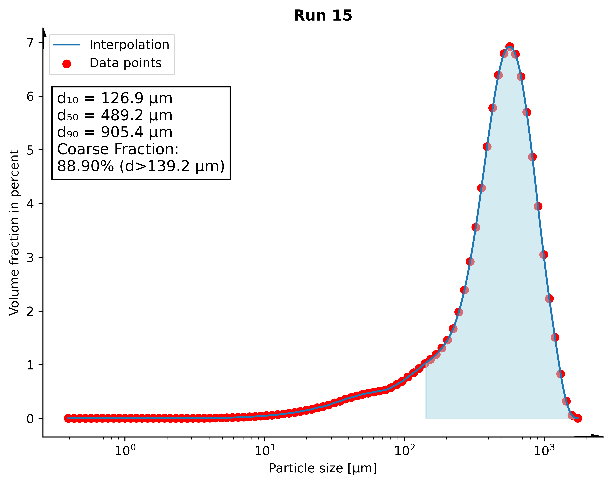 |
| 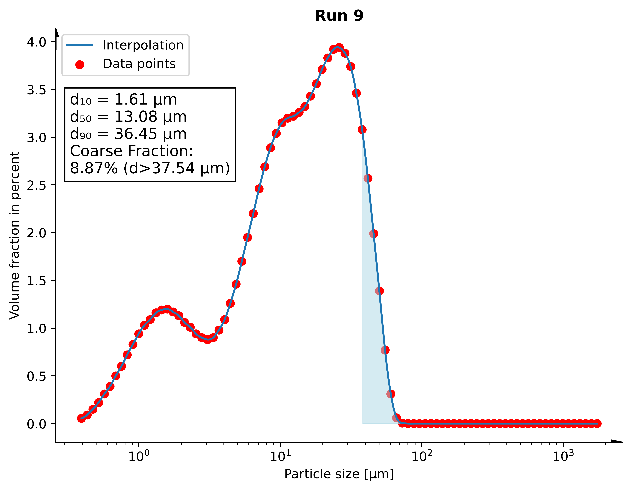 | 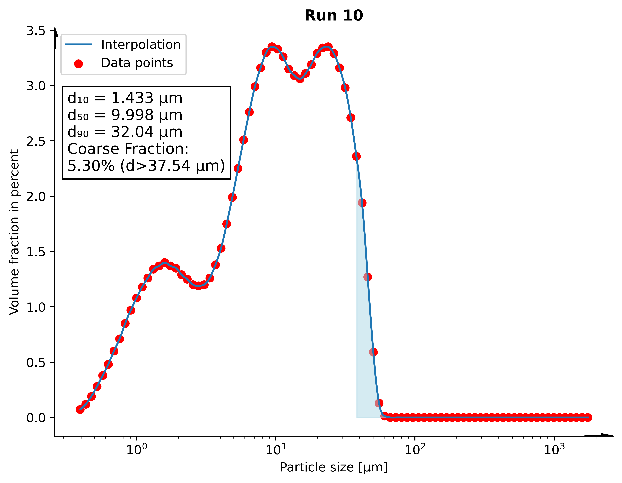 |
| 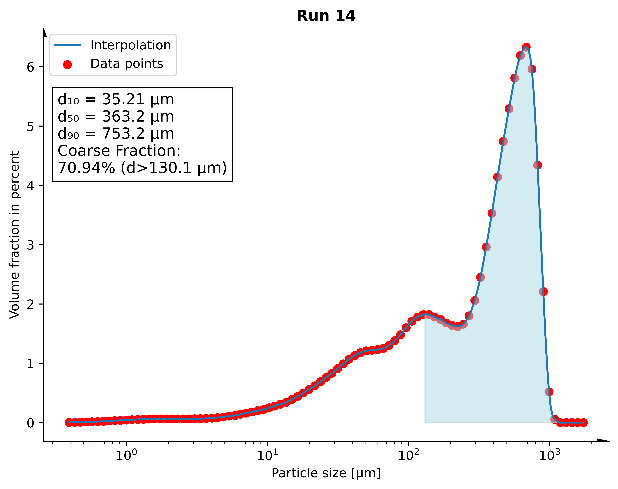 | 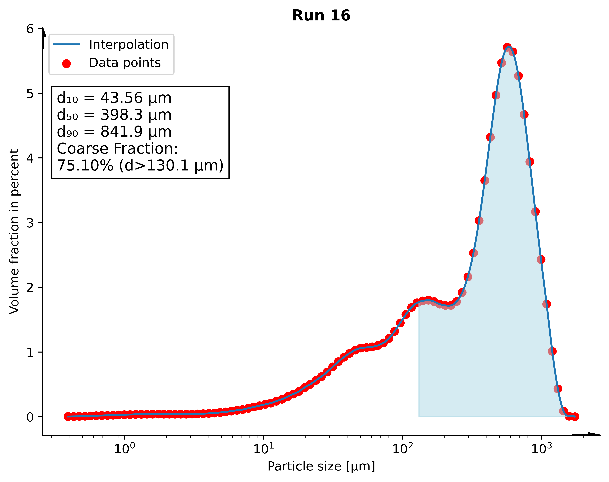 |

*Figure S 4 PSD of the granules of the replicates performed following the experimental mixture design; Granules of run 4 and 17 are based on 100 % MCC; Granules of run 5 and 15 are based on 100 % SMCC; Granules of 9 and 10 are based on 100 % DCP; Granules from run 14 and 16 are based on a ternary mixture of 33 % DCP, 33 % MCC, and 33 % SMCC; areas in blue correspond to particle sizes > d_90_ of the related physical mixtures (concrete numbers are given in brackets).*

| **a**  **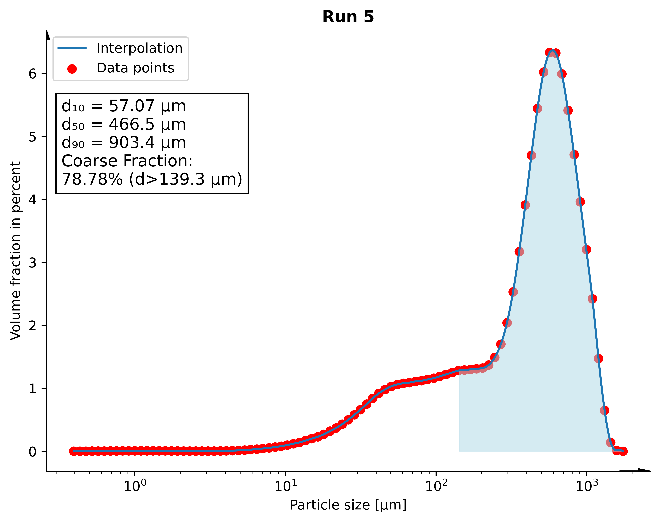** | **b**  **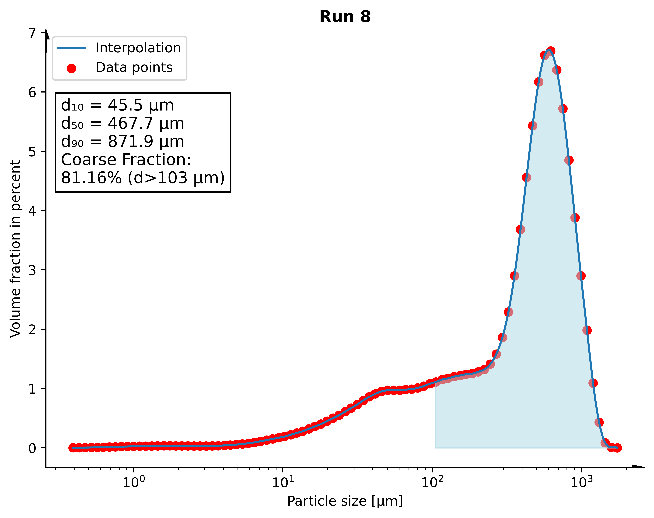** |
| --- | --- |
| **c**  **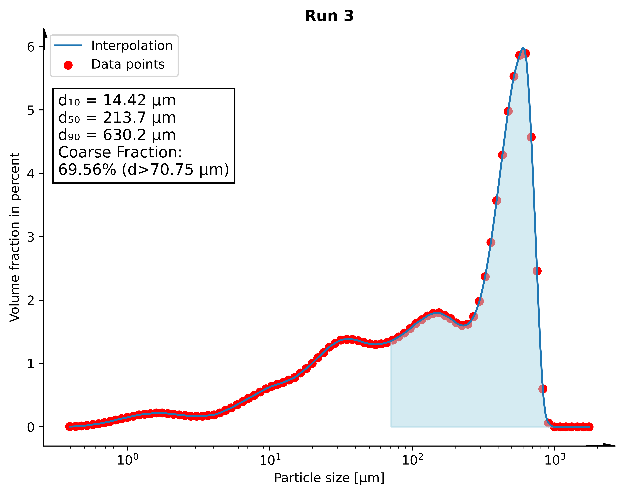** | **c**  **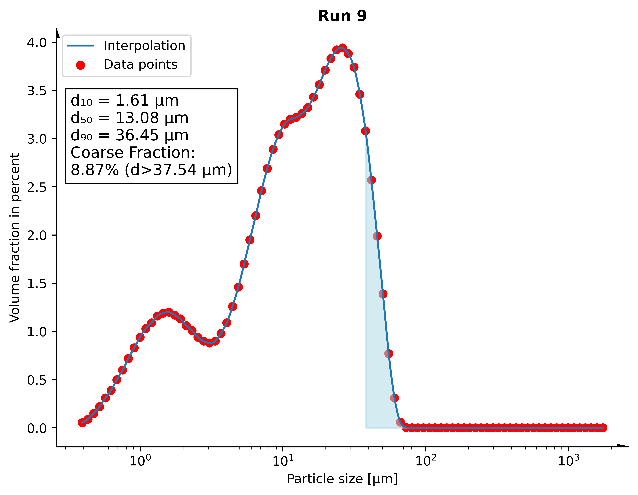** |

*Figure S 5 PSD of granules with SMCC from (a) to (d), whereby (a) displays the results of granules based on 100 % SMCC with 0 % DCP; (b) displays the results of granules based on 67 % SMCC; (c) displays the results of granules based on 33 % SMCC; (d) displays the results of granules based on 0 % SMCC with 100 % DCP.*

| 1. **Fines** | 1. **d50** |
| --- | --- |
| 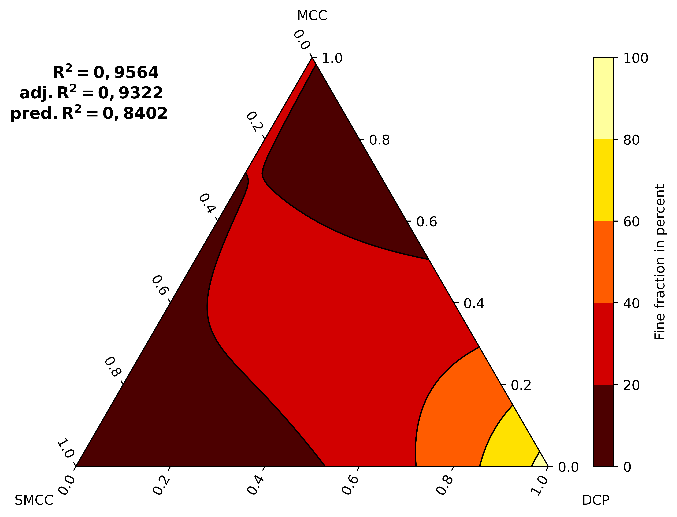 | 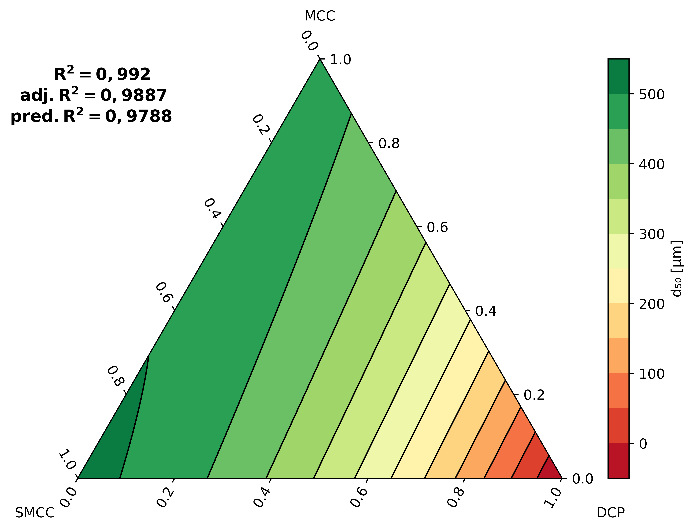 |
| 1. **Hausner Ratio** |  |
| **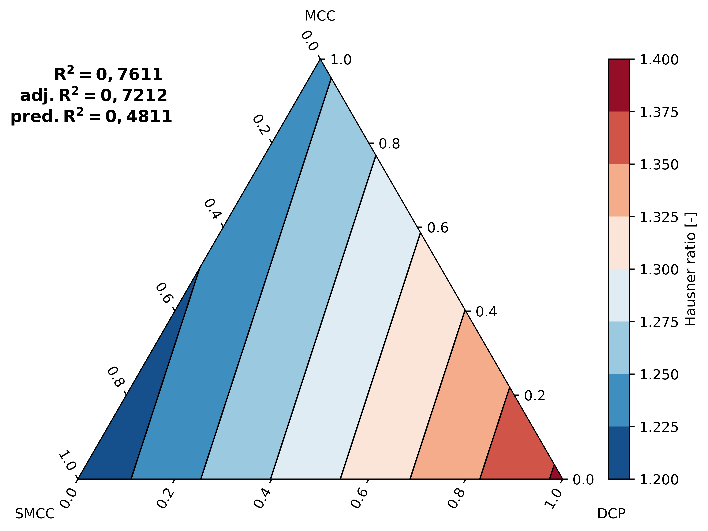** |  |

*Figure S 6: Contour plots of the mixture design models for the fines (a), defined as all particles <d_90_ of the related physical mixture, the d_50_ value (b), and the Hausner Ratio (b).*
